# Supplementary material for: Differences in the Response to DNA Double-Strand Breaks between Rod Photoreceptors of Rodents, Pigs, and Humans
Source: Cells. 2020 Apr 12;9(4):947. doi: 10.3390/cells9040947 (PMC7226979; doi:10.3390/cells9040947)
Supplement: Supplementary file 1 [file cells-09-00947-s001.pdf]

Supplementary Figures

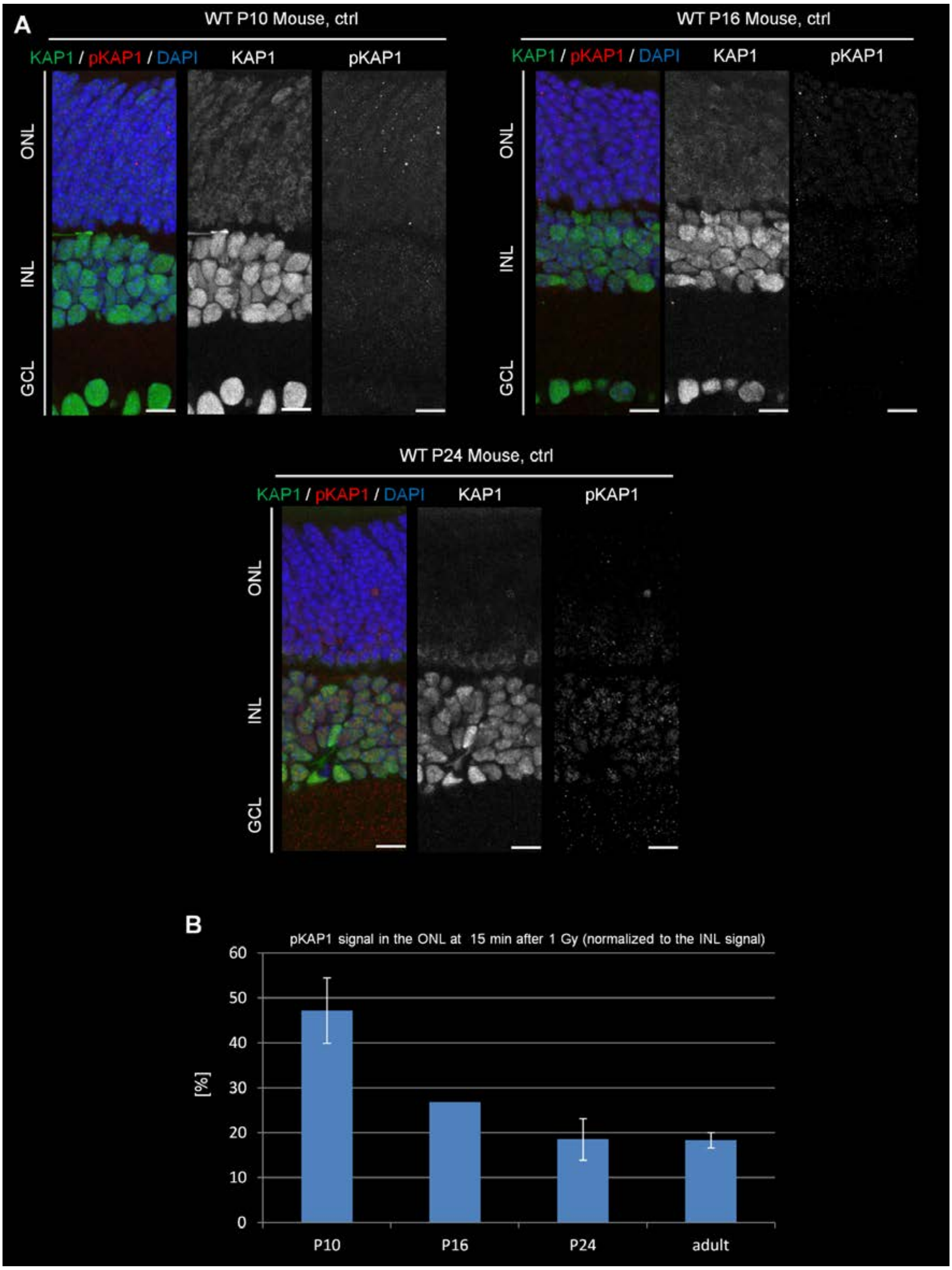

Figure S1: Staining and quantification of pKAP1 signals in the ONL of Lbr-TER mice. (A) Immunofluorescence images of KAP1 (green) and pKAP1 (red) at 15 min after 1Gy in the retinae of P10, P16 and P24 ctrl mice. Nuclei were counterstained with DAPI (blue). Scale bars represent 15 µm. (B) Quantification of the pKAP1 fluorescence in the ONL of P10, P16, P24 and adult WT mice was measured at 15 min after 1 Gy. The signal intensity in the ONL was normalized to the signal intensity in the INL. Background signal intensities were subtracted. All quantitative data are presented as mean ± SEM from 2 (P10, P24 and adult) or 1 experiment (P16).

1

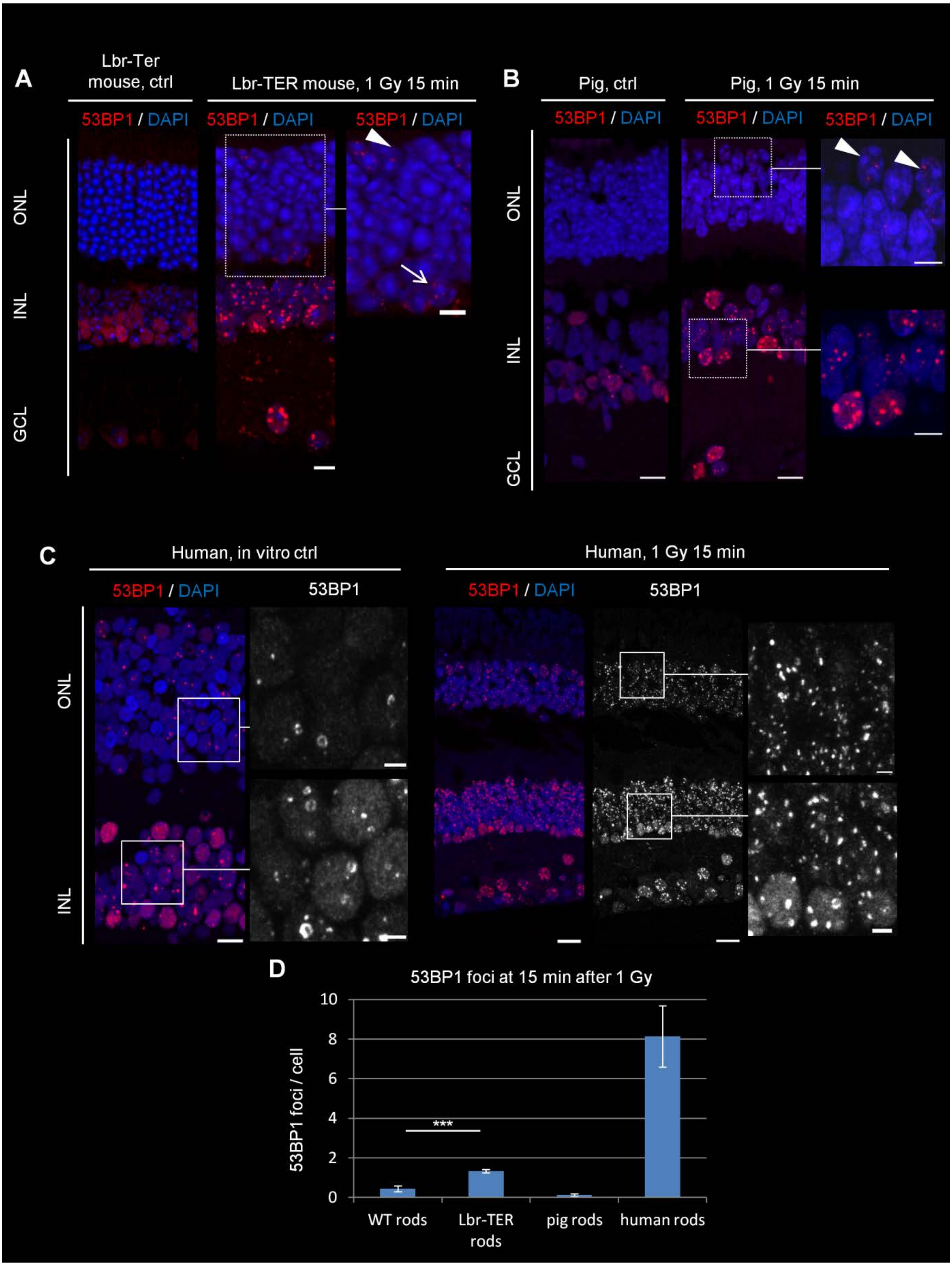

A
Lbr-Ter mouse, ctrl
Lbr-TER mouse, 1 Gy 15 min
53BP1 / DAPI
ONL
INL
GCL
B
Pig, ctrl
Pig, 1 Gy 15 min
C
Human, in vitro ctrl
Human, 1 Gy 15 min
53BP1
D
53BP1 foci at 15 min after 1 Gy
53BP1 foci / cell
***
WT rods
Lbr-TER rods
pig rods
human rods

Figure S2: 53BP1 expression and its focus formation after IR in the retinae of Lbr-TER mice, pigs and humans. (A) Immunofluorescence images of 53BP1 (red) in retinae of ctrl and irradiated Lbr-TER mice at 15 min after 1Gy. Nuclei were counterstained with DAPI (blue). Scale bars represent 20 µm in whole retina pictures and 5 µm in ONL and INL magnifications. Arrowheads mark cones and arrows mark Lbr-TER rods that both show slightly increased 53BP1 foci formation when compared to WT rods. (B) Immunofluorescence images of 53BP1 (red) in retinae of ctrl and irradiated pigs at 15 min after 1Gy. Nuclei were counterstained with DAPI (blue). Scale bars represent 15 µm in whole retina pictures and 5 µm in ONL and INL magnifications. Arrowheads mark cones that show 53BP1 foci formation. (C) Immunofluorescence images of 53BP1 (red) in an unirradiated human retinal explant and one explant at 15 min after 1 Gy. Nuclei were counterstained with DAPI (blue). Scale bars represent 10 µm in whole retina pictures and 2.5 µm in ONL and INL magnifications. (D) Quantification of IR-induced 53BP1 foci in rods of humans and pigs as well as in WT and Lbr-TER rods of Lbr-TER mice at 15 min after 1 Gy. Quantitative data are presented as mean ± SEM from three independent retinal explants for humans and three different animals for Lbr-TER mice and pigs. p values were obtained by t test and represent a comparison of all cells analyzed in the indicated cell population (at least 20 cells for humans and pigs and 10 cells for Lbr-TER mice) with ***: $p < 0.001$.

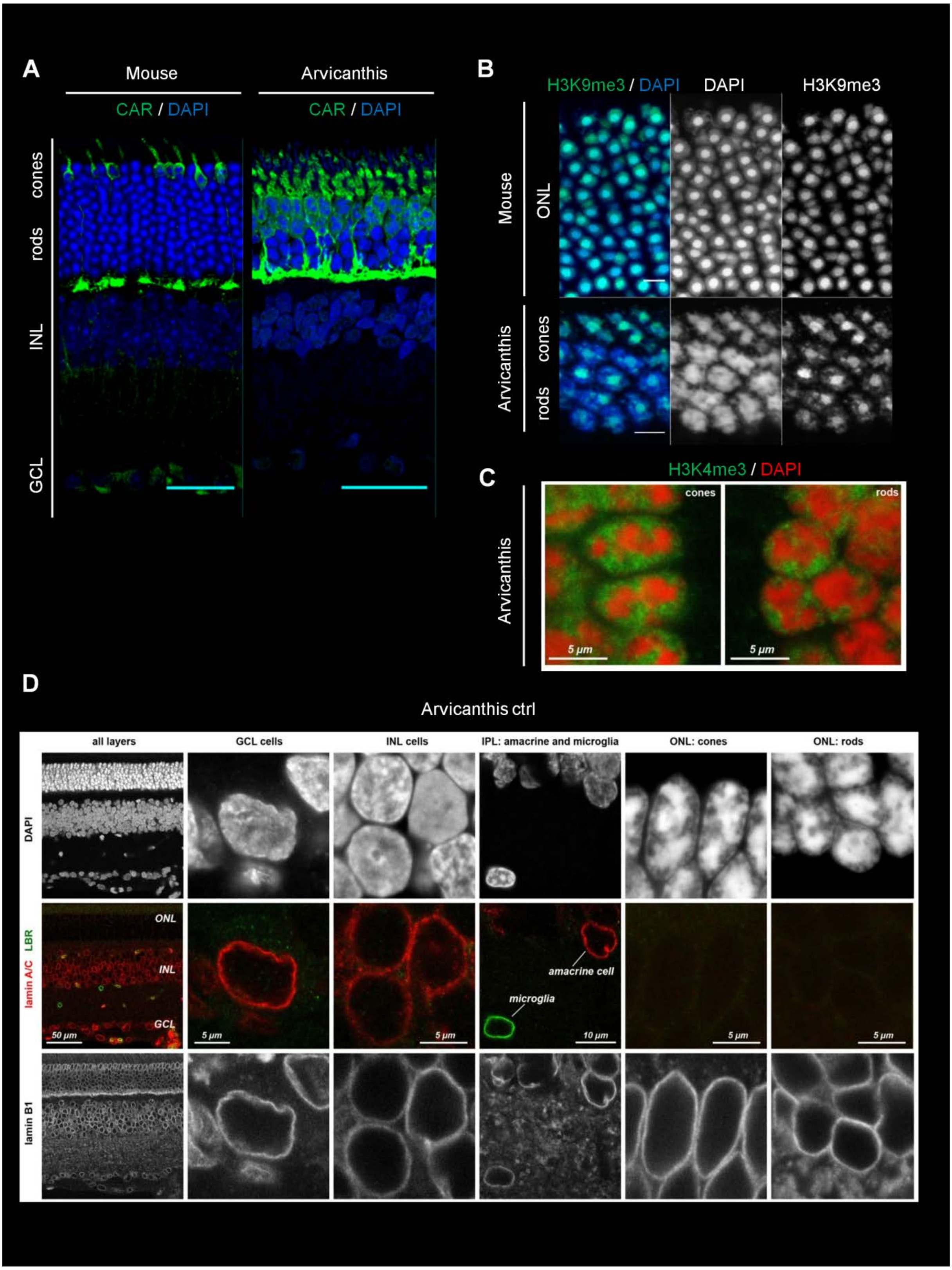

A
Mouse
Arvicanthis
CAR / DAPI
CAR / DAPI
cones
rods
INL
GCL
B
H3K9me3 / DAPI
DAPI
H3K9me3
Mouse
ONL
Arvicanthis
cones
rods
C
H3K4me3 / DAPI
Arvicanthis
cones
rods
5 µm
5 µm
D
Arvicanthis ctrl
all layers
GCL cells
INL cells
IPL: amacrine and microglia
ONL: cones
ONL: rods
DAPI
lamin A/C LBR
ONL
INL
GCL
50 µm
5 µm
5 µm
amacrine cell
microglia
10 µm
5 µm
5 µm
lamin B1

1

2

Figure S3: Lack of LBR and lam A/C expression in the inverted nuclei of rod PRs in Arvicanthis ansorgei. (A) Cones in the retinae of mice and Arvicanthis identified by immunofluorescence staining against cone arrestin (CAR, green). Nuclei were counterstained with DAPI (blue). Scale bars represent 10 µm. Please note much higher numbers of cone PRs in the retina of Arvicanthis when compared to the mouse retina. (B) Immunofluorescence images of the heterochromatin marker $H_3K_9me_3$ (green) in PRs of adult mice and Arvicanthis. Nuclei were counterstained with DAPI (blue). Scale bars represent 5 µm. (C) Immunofluorescence images of the euchromatin marker $H_4K_3me_3$ (green) in PRs of adult Arvicanthis. Nuclei were counterstained with DAPI (red). Scale bars represent 5 µm. Please note that the heterochromatin marker $H_3K_9me_3$ stained centrally located heterochromatin clusters in both rods and cones of Arvicanthis, similar to the situation in rods of mice. In contrast, euchromatic $H_3K_4me_3$ signals were located in the periphery of both PR types of Arvicanthis. Thus, the analysis of chromatin distribution clearly shows an IoC in both rods and cones of Arvicanthis. (D) Immunofluorescence images of lam A/C (red), LBR (green) and lamin B1 (gray, lowest lane) in the retina of adult Arvicanthis. Nuclei were counterstained with DAPI (gray, upper lane). Please note that neither lam A/C nor LBR is expressed in rod and cone PRs of Arvicanthis.

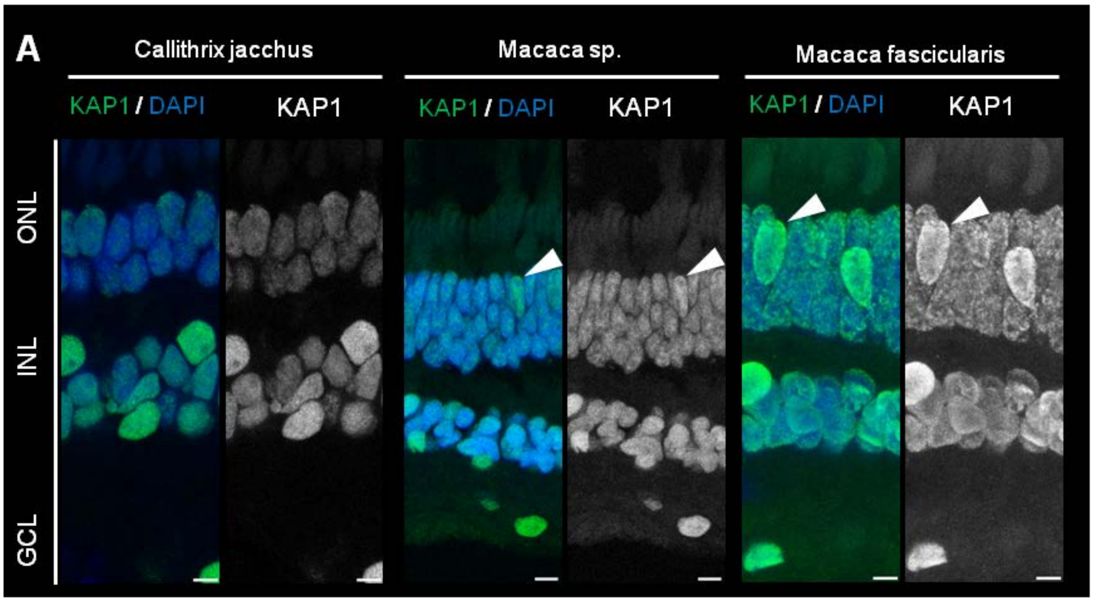

Figure S4: KAP1 expression in the retinae of primates. (A) Immunofluorescence images of KAP1 (green) in the retinae of Callithrix jacchus, Macaca sp. and Macaca fascicularis. Nuclei were counterstained with DAPI (blue). Arrowheads are marking cone PRs. Scale bars represent 10 μm.

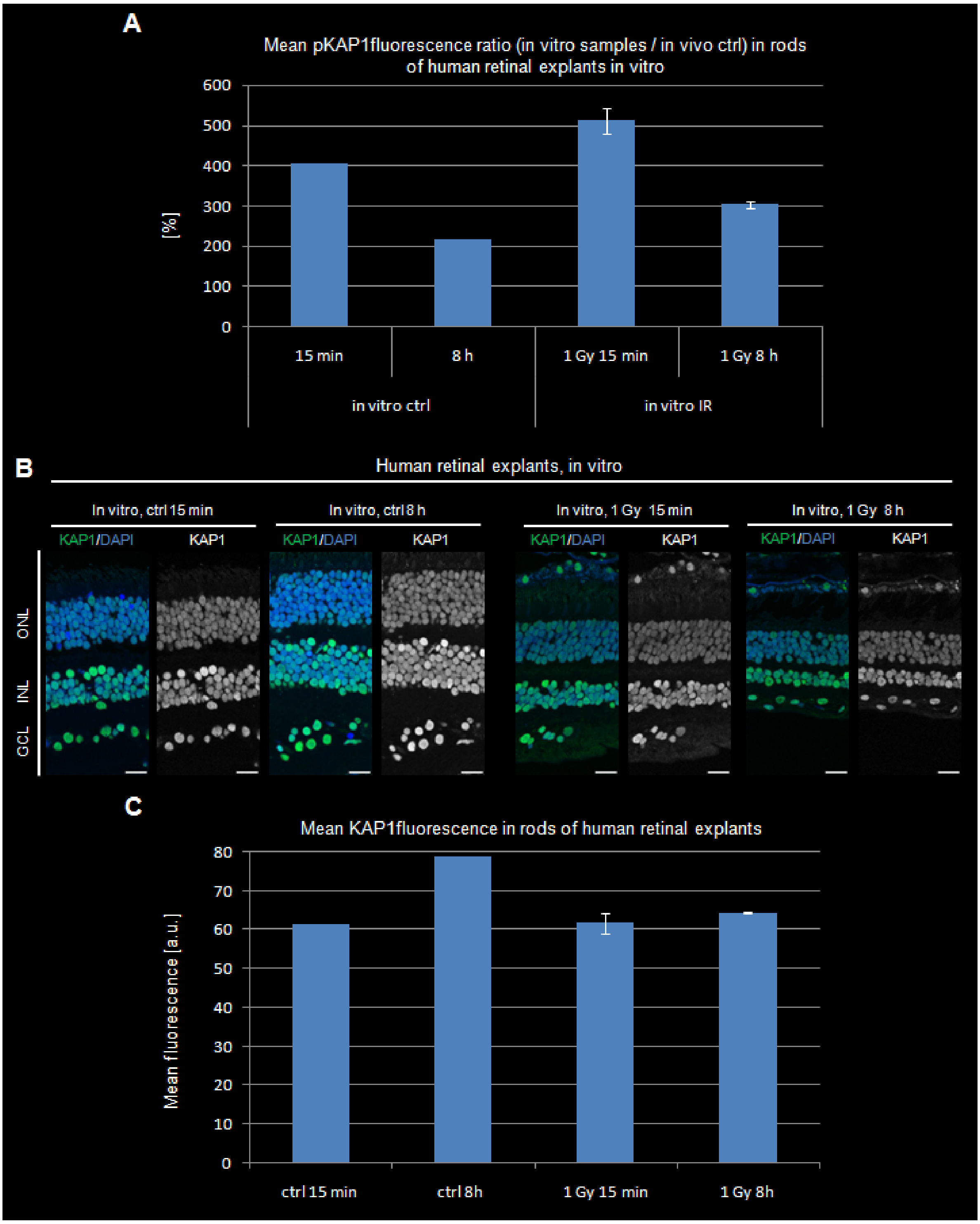

Figure S5: Quantification of KAP1 and pKAP1 levels in rods of human retinal explants. (A) Quantification of the pKAP1 signals in the rod nuclei of human retinal ctrl explants and irradiated samples at different time points after in vitro irradiation with 1 Gy. Fluorescence values were normalized to the value in rods of the in vivo control sample. (B) Immunofluorescence images of KAP1 (green) in the various retinal explants that were taken for the in vitro experiment. Nuclei were counterstained with DAPI (blue). Scale bars represent 20 µm. (C) Quantification of the KAP1 signals in human retinal ctrl explants and irradiated samples at different time points after in vitro irradiation with 1 Gy. Please note that there are no big differences in KAP1 signal levels that might explain the differences measured in pKAP1 signal levels presented in (A). Data are presented as mean ± SEM from analysis of 1 to 3 retinal explants for each time points (for ctrls n= 1, for irradiated samples n=3).
